# Chemicals in plastics differentially affect the transcriptome of MCF-7 breast cancer cells

**DOI:** 10.1007/s00204-026-04376-1

**Published:** 2026-04-20

**Authors:** Geronimo Matteo, David C. Eickmeyer, Lauren M. Bradford, Matthew J. Meier, Andrew Williams, Tara Barton-Maclaren, J. Christopher Corton, Carole L. Yauk, Ella Atlas

**Affiliations:** 1 Environmental Health Science and Research Bureau, Health Canada, Ottawa, Canada; 2 Department of Biology, University of Ottawa, Ottawa, Canada; 3 Existing Substances Risk Assessment Bureau, Ottawa, Canada; 4 Office of Chemical Safety and Pollution Prevention, US Environmental Protection Agency, Research Triangle Park, USA; 5 Department of Biochemistry, University of Ottawa, Ottawa, Canada

**Keywords:** Plastic chemicals, Risk assessment, Transcriptomics, Endocrine disruptors, BPA

## Abstract

Humans are exposed to chemicals that leach from plastics, yet many remain data-poor and lack toxicological evaluation. High-throughput transcriptomics (HTTr) provides a scalable way to screen chemicals and generate mechanistic insights relevant to human health risk assessment. We applied HTTr in MCF-7 breast cancer cells to assess chemicals across several concentrations (0.001–50 µM) used in plastics and dyes. Transcriptomic points of departure (tPODs) were derived from general gene perturbations, including pathway-level and estrogen receptor α (ERα)–specific changes. We also used transcriptomic biomarkers to evaluate ERα activity and cellular stress responses. Most plastic chemicals showed similar toxicological potency, with tPODs falling within one order of magnitude. Bisphenol K was the most potent, activating ERα at the lowest concentrations, followed by plastic additive 08 and bisphenol A (BPA). Despite similar overall potency, transcriptomic biomarkers revealed distinct mechanisms. Chemicals structurally similar to BPA activated ERα, whereas others with different functional groups inhibited the ERα biomarker. Pathway and upstream regulator analyses further indicated that BPA-like chemicals consistently perturbed ERα-related pathways, while other chemicals enriched fewer gene sets and often produced opposite directional responses. At the highest concentrations tested, several chemicals also activated stress-response biomarkers and suppressed proliferation. Overall, these results suggest that while many plastic chemicals exhibit comparable in vitro potency, they diverge in ERα regulation and downstream biological pathways. This study demonstrates the reproducibility and value of HTTr for chemical screening, supports grouping ERα-active plastic chemicals for read-across, and underscores the need for additional evaluation of plastic chemicals.

**Supplementary Information:**

The online version contains supplementary material available at 10.1007/s00204-026-04376-1.

## Introduction

Global plastic production exceeds 400 million tons annually (Houssini et al. 2025), making plastics among the most pervasive materials in modern society. More than 16,000 plastic chemicals are known, with about 4200 recently identified as chemicals of concern due to their environmental persistence, potential for bioaccumulation, and possible toxicity (Monclús et al. 2025). Plastic additives are a subset of chemicals incorporated into plastics to improve performance of materials. The additives can increase flexibility (plasticizers), stability against heat and light (stabilizers), resistance to microbial growth (biocides), reduce flammability (flame retardants), provide colour (colourants/dyes), and more (Pfaendner 2019). Many chemicals in plastics are not covalently bound to the core polymer and thus can leach from plastic materials contributing to environmental pollution (Hahladakis et al. 2018; Zhang et al. 2024b). While some classes like bisphenols and phthalates are relatively well studied, most plastic chemicals remain poorly characterized. Thus, there is limited toxicological data, and few have been screened for potential endocrine-disrupting effects, limiting the understanding of the potential for these chemicals to pose a risk to human health or the environment throughout their lifecycle.

One of the most studied chemicals used in plastics production is bisphenol A (BPA), used mainly in polycarbonate plastics and epoxy resins, as well as in some materials as a developing agent and antioxidant (Hahladakis et al. 2018). Humans are exposed to BPA through food packaging (Manzoor et al. 2022) and thermal paper such as receipts (Reale et al. 2021). Despite its short half-life (< 6 h; (Thayer et al. 2015), BPA is consistently detected in urine, indicating continuous exposure and widespread presence in the environment (Adamovsky et al. 2024). Due to its endocrine disrupting effects, BPA has been restricted in products like baby bottles in some jurisdictions, leading to the proliferation of BPA substitutes that are increasingly detected in biomonitoring studies (Wang et al. 2023), although many remain poorly characterized.

BPA interacts with nuclear hormone receptors including estrogen receptor α (ERα). ERα is expressed in hormone sensitive tissues like the mammary gland, where it plays an integral role in health and disease (Chen et al. 2022). Most breast cancers overexpress ERα (Clusan et al. 2023) and chemicals that interact with this receptor may increase breast cancer risk (Gonzalez et al. 2019). BPA has been detected in breast adipose tissue of breast cancer patients (Reeves et al. 2018), and its urinary levels have been positively associated with breast cancer risk (Keshavarz-Maleki et al. 2021). Further, there is mounting evidence that plastic chemicals that share a similar chemical structure to BPA activate the ERα in vitro (Mesnage et al. 2017; Pelch et al. 2019; Matteo et al. 2023, 2025; Beal et al. 2024) and negatively impact mammary development and function in vivo (Vandenberg 2021). For this work, we nominally categorized BPA-like chemicals as those containing a bisphenol structure and two para-substituted hydroxyl groups.

New approach methods (NAMs) are increasingly being used to evaluate chemicals for human health hazards as part of regulatory assessments (Health Canada 2025). In vitro systems like MCF-7 breast cancer cells that overexpress the ERα are used to screen chemicals for estrogenic and toxicological effects. Our lab (Matteo et al. 2023, 2025) and others (Mesnage et al. 2017; Harrill et al. 2024; Beal et al. 2024) have used high throughput transcriptomics (HTTr) to screen data-poor chemicals in plastics for estrogenic effects in MCF-7 cells. These approaches have relied on deriving measures of transcriptomic activity and ERα-specific toxicological potencies as well as applying transcriptomic biomarkers. Several studies support the hypothesis that many BPA-like plastic-related chemicals activate the ERα at similar concentrations as BPA, share similar mechanisms of action, and behave additively when present as mixtures (Matteo et al. 2023, 2025; Beal et al. 2024).

We screened nine plastic-related chemicals, for which there is limited toxicity data, using HTTr in the MCF-7 breast cancer cell model. Bisphenol E (BPE), diallyl BPA (DA-BPA), bisphenol M (BPM), bisphenol K (BPK), tris(4-hydroxyphenyl)ethane (THPE), antioxidant 425 (AO425), antioxidant 2246 (AO2246), plastic additive 08 (PA08), and tetrabromo bisphenol S (TBBPS), are used in plastic materials for various applications, in addition to other uses. In addition, we included two dyes (phenol red; PRed; tetrabromophenol blue; TBPB) commonly used in laboratory settings and in some consumer products, as well as an antimicrobial (bithionol; BTH), that are suspected of possible endocrine disrupting effects. MCF-7 cells were exposed to chemicals across eight concentrations (0.001–50 µM) for 48 h, as well as BPA, a solvent control (0.1% DMSO) and a positive control (17β-estradiol, 0.1 and 1 nM). General transcriptomic responses were measured using Templated Oligonucleotide Sequencing (TempO-Seq™; BioSpyder Inc.). ERα activation and cellular stress responses were evaluated using transcriptomic biomarkers. Benchmark concentration (BMC) modeling was applied to gene expression data to derive general, ERα-specific, and pathway-level transcriptomic points of departure (tPODs) for transcriptomic changes. In addition, genes with modeled BMCs were analyzed for pathway enrichment and upstream regulator activity to explore mechanisms of toxicity. The overall aim of this study was to rank these chemicals based on their toxicological potency and to identify chemicals with potential estrogenic properties.

## Methods and materials

### Chemicals

**Fig. 1:**
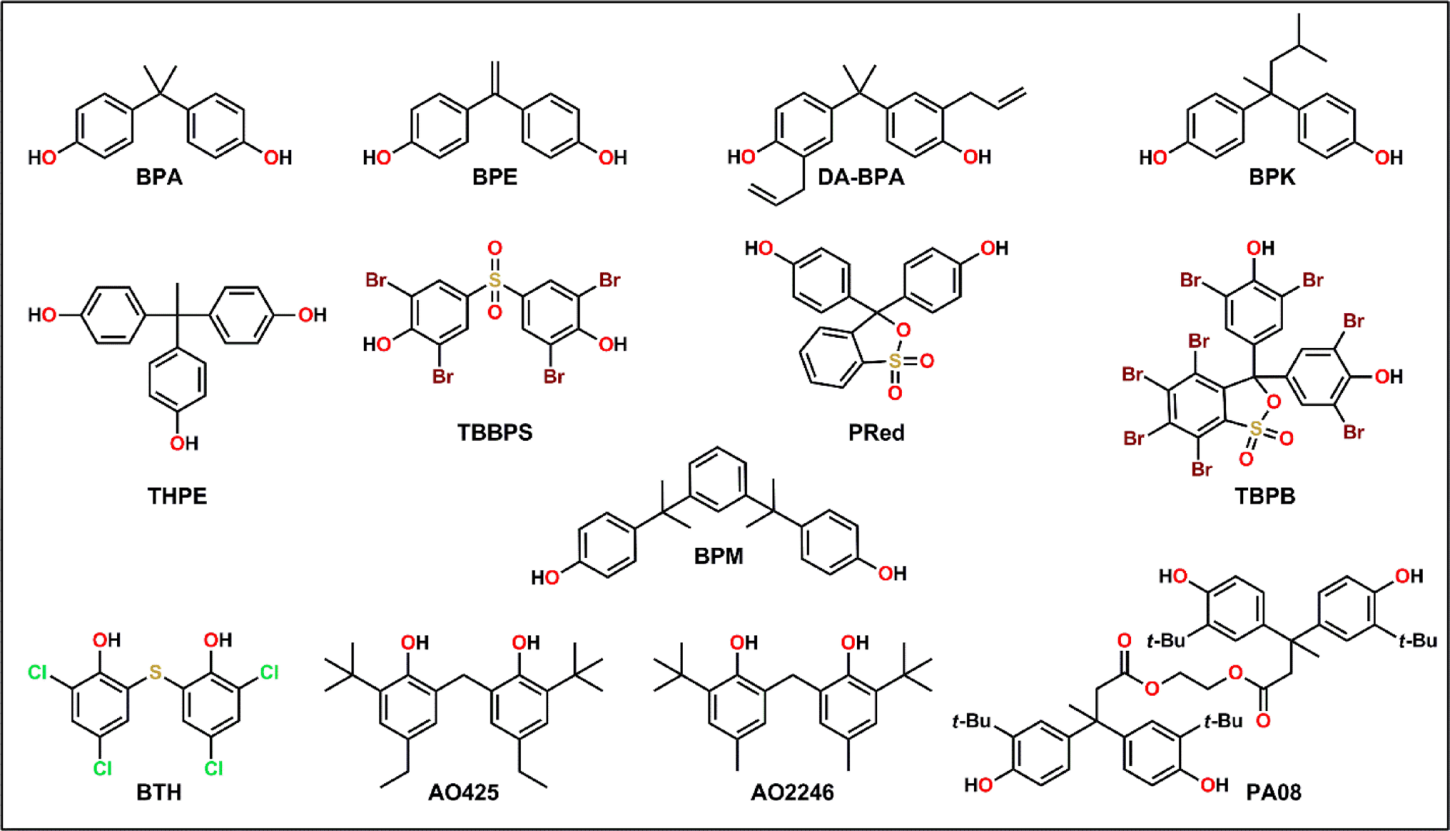
Chemical structure of plastic chemicals and dyes tested

Table 1 lists the chemicals used and their respective suppliers. All chemicals (Table 1, Fig. 1) were dissolved in dimethyl sulfoxide (DMSO) to achieve a final DMSO concentration of 0.1% in exposure media. We note that commercially available Hostanox® 03 is referred to as PA08 in this manuscript.

**Table 1:** List of chemicals and suppliers

CAS RN	Chemical	Supplier	Molecular weight (g/mol)
50-28-2	Estradiol (E2)	Sigma-Aldrich Inc (St. Louis, MO, USA)	272.4
67-68-5	Dimethyl sulfoxide (DMSO)		78.14
29348	Bisphenol A (BPA)		228.29
2081-08-05	Bisphenol E (BPE)		214.26
1745-89-7	Diallyl BPA (DA-BPA)		308.4
88-24-4	Antioxidant 425 (AO425)		368.6
13595-25-0	Bisphenol M (BPM)	Toronto Research Chemicals (Vaughan, ON, Canada)	346.5
4430-25-5	Tetrabromophenol Blue (TBPB)		985.5
32509-66-3	Plastic additive 08 (PA08)		795.1
143-74-8	Phenol Red (PRed)		354.4
119-47-1	Antioxidant 2246 (AO2246)	Dr. Ehrenstorfer (Augsburg, Germany)	340.5
6807-17-6	Bisphenol K (BPK)		270.4
39635-79-5	Tetrabromo BPS (TBBPS)		565.9
97-18-7	Bithionol (BTH)		356
27955-94-8	Tris(4-hydroxyphenyl)ethane (THPE)		306.4

### Cell culture

MCF-7 cells (ATCC, Manassas, Virginia) were cultured in 10 cm dishes (Corning Falcon No. 353003, Corning, New York) with Dulbecco’s modified Eagle’s medium (DMEM; Gibco, Thermo Fisher Scientific, Waltham, Massachusetts) containing 10% fetal bovine serum (FBS; Wisent Bioproducts, Saint-Jean Baptiste, QC, Canada), and 1% penicillin and streptomycin (P/S; Wisent), at 37 °C in a humidified atmosphere (5% CO_2_). For the experiments, cells were washed with phosphate-buffered saline (PBS) and the media were replaced with phenol red-free DMEM supplemented with 5% charcoal–dextran stripped FBS (CD-FBS) to eliminate estrogens present in commercial media and serum (Atlas et al. 2003) for 24 h. Cells were then seeded into clear 96-well plates (Corning Falcon No. 353075) at a density of 2.0 × 10^4^ cells per well in phenol red-free DMEM supplemented with 5% CD-FBS and 1% P/S. After 24 h, the medium was removed and replaced with phenol red-free DMEM containing 5% CD-FBS and 1% P/S and chemicals of interest. Cells were exposed for 48 h, in quadruplicate, to BPA and the 12 chemicals of interest at eight different concentrations (0.001, 0.01, 0.1, 0.5, 1, 5, 10, 50 µM), 17β-estradiol (E2; 0.1, 1 nM) as a positive control, as well as at least four plate-matched solvent controls (0.1% DMSO). These concentrations span the approximate ranges reported for BPA in human fluids (Health Canada 2021; Colorado-Yohar et al. 2021). The exposure timeframe was selected based on previous studies by our lab (Matteo et al. 2023, 2025). After exposure, cells were washed with PBS and lysed in situ using 2 × TempO-Seq lysis buffer diluted with an equal amount of PBS for TempO-Seq library building.

### Cell viability assay

To determine if chemical exposure decreased cell viability, MCF-7 cells were seeded into black 96-well plates (Corning Falcon No. 3603) and treated as described above. Cell viability was measured using the CellTiter-Blue Cell Viability Assay (Promega Corp, Madison, Wisconsin), as per the manufacturer’s instructions. After 48 h chemical exposure, CellTiter-Blue reagent was added to each well, and the plates were incubated (37 °C, 5% CO_2_) for 2 h. The fluorescence was read using an excitation wavelength of 560 nm and emission of 590 nm using a SpectraMax M2 (Molecular Devices LLC, San Jose, California). Fluorescence readings were expressed as a percent ratio to that of the DMSO control group and cytotoxicity was defined as readings < 50% of the control.

### TempO-Seq™ library building and next-generation sequencing

Gene expression was measured using the TempO-Seq™ Human Whole Transcriptome v2.1 kit (BioSpyder Technologies Inc, Carlsbad, California) as per the manufacturer’s instructions and as previously described (Matteo et al. 2023, 2025). Cell lysates and positive technical controls (Human Universal Reference RNA—uhrRNA Agilent Cat No. 740000, Santa Clara, California, and Human Brain Total RNA brRNA—ThermoFisher AM7962, Waltham, Massachusetts), as well as no-cell negative controls (1 × TempO-Seq™ lysis buffer) were hybridized to the Detector Oligo (DO) Pool using an annealing kit for the whole human genome supplied by BioSpyder. The hybridization mixture was incubated for 10 min at 70 °C followed by a temperature gradient with a ramp rate of 0.5 °C/min to 45 °C over 50 min with a 16-h hold at 45 °C and then cooled to 25 °C. Nuclease digestion was employed to remove excess, unbound, or incorrectly bound DOs at 37 °C for 90 min. Amplification templates were generated by ligating DO pairs bound to adjacent target sequences for 1 h at 37 °C, followed by enzyme denaturation for 15 min at 80 °C. Amplification templates (10 µL) were pipetted into a 96-well PCR Pre-Mix and Primer plate supplied by BioSpyder and amplified using a CFX96 Real-Time PCR Detection System (Bio-Rad, Hercules, California) to incorporate a unique sample index/tag sequence and the sequencing adaptors for each sample. The following PCR settings were used: 37 °C for 10 min, 95 °C for 1 min; 25 cycles of 95 °C for 10 s, 65 °C for 30 s, 68 °C for 30 s (with optical read for visual sample quality control [QC]); 68 °C for 2 min; and hold at 25 °C prior to library pooling and purification.

NucleoSpin Gel and PCR Clean-up kits (Macherey–Nagel, Dueren, Germany) were used to pool and purify labeled amplicons. Next-generation sequencing libraries were sequenced using a NextSeq 2000 High-Throughput Sequencing System (Illumina, San Diego, California), using 50 cycles from a 100-cycle high-throughput flow cell to achieve a median read depth of 2 million reads per sample. Data were processed as described below and reads were aligned to the BioSpyder TempO-Seq™ Human Whole Transcriptome probe set version 2.0 (22 537 probes over 19 687 genes).

### Data processing and quality control

Reads were demultiplexed from the BCL files and processed into FASTQ files using DRAGEN analysis v1.3.0 on the Illumina BaseSpace Sequence Hub (Illumina, Inc., San Diego, CA, USA). Processing and quality control of the FASTQ files, differential expression analysis, and exploratory statistical analyses were performed using the R-ODAF_Health_Canada pipeline (https://github.com/R-ODAF/R-ODAF_Health_Canada; accessed December 2024), which manages workflows using snakemake v8.0 (Mölder et al. 2021) and relies on scripts in R (v.4.3.2). The preprocessing steps use fastp v0.23.2 for trimming (Chen et al. 2018), STAR v2.7.8a (Dobin et al. 2013) to perform alignment of raw reads to the reference sequence and the qCount function of the QuasR R package (v1.30.0; (Gaidatzis et al. 2015) to extract the feature counts from the aligned reads (BAM files) using features specified in a GTF file. A samples-by-probes count matrix was produced. Study-wide quality control was performed on the count matrix using several methods to measure consistency and remove low-quality samples as a guideline (Harrill et al. 2024). A cut-off of 0.1 for 1-Spearman’s ρ was used to remove samples that were not correlated with others in this study. We also used a 10% cut-off of uniquely mapped reads as the number of target sequences (e.g., 100 000 reads to pass filter when the target is 1 000 000 for TempO-Seq experiments) as previously described (Harrill et al. 2024). We removed any samples outside of Tukey’s Outer Fence (3 × interquartile range) for: (1) the number of probes capturing the top 80% of the signal, and (2) the number of detected probes (those with at least 5 mapped reads). Samples with a Gini coefficient (a measure of inequality in distributions) > 0.95 were excluded. Samples removed by these criteria are listed in Supplementary File 1.

To create a matrix for biomarker analysis, individual pairwise contrasts for each concentration and each chemical tested were created to the respective 0.1% DMSO control samples for each plate. Following the recommendations set out by the Omics Data Analysis Frameworks for Regulatory application (R-ODAF) guidelines (Verheijen et al. 2022), genes were filtered for each contrast tested to include only those where 75% of at least one experimental group were above 0.5 counts per million (CPM), and spurious spikes were removed in which [max–median] of counts were less than [sum of counts]/[number of replicates + 1]. We used DESeq2 1.30.0 (Love et al. 2014) to estimate fold changes and normalize for library size within the TempO-Seq™ data. The ashr method was used to perform log2 fold change shrinkage (Stephens 2017).

### Gene expression biomarker analysis

To determine if the chemicals activate the ERα or other pathways, the expression profile of each chemical-concentration tested relative to solvent control (derived from the analysis of gene sets with unadjusted *p* value < 0.05 and absolute linear fold change ≥ 1.2) was compared to several characterized biomarkers as previously described (Kupershmidt et al. 2010). The biomarkers included those that predict modulation of ERα (Corton et al. 2024b), nuclear factor erythroid 2-related factor 2 (NRF2; Rooney et al. 2020), heat shock factor 1 (HSF1: Cervantes and Corton 2021), metal-induced transcription factor 1 (MTF1; Jackson et al. 2020), DNA damage (TGx-DDI; Li et al. 2019), histone deacetylase inhibition (TGx-HDACi; Cho et al. 2021), nuclear factor kappa B (NF-κB; Korunes et al. 2022), hypoxia inducible factor 1 (HIF1α; Corton et al. 2025), and cellular proliferation (Corton et al. 2024a). We also included an unpublished biomarker that predicts unfolded protein response mediated by the X-box binding protein 1 (XBP1; manuscript in preparation). The correlations between each biomarker and the gene lists of chemicals were determined using the Running Fisher algorithm in the BaseSpace Correlation Engine as described previously (Ryan et al. 2016). The Running Fisher algorithm provides an assessment of the statistical significance of the correlation of the overlapping genes between the biomarker and each gene list. A complete description of the Running Fisher test has been published (Kupershmidt et al. 2010). The results were exported, and each p-value was converted to a − log(*p* value). Thresholds for significance were set at − log(*p* value) ≥ 4 for activation or ≤  − 4 for suppression based on prior studies using this threshold (Matteo et al. 2023, 2025). As in our previous work (Matteo et al. 2023, 2025), we considered activation of at least two cellular stress response biomarkers (NRF2, HSF1, MTF1, TGx-DDI, NF-kB, HIF1α) within a condition as an indication of overt cellular stress (Escher et al. 2020) and thus filtered out these concentrations from subsequent BMC analyses.

### Transcriptomic point of departure analysis

BMC modeling was done as described previously (Matteo et al. 2023, 2025) in BMDExpress3. A Williams trend test (*p* < 0.05) and absolute fold change ≥ 1.5 was applied to filter probes. Best fit models from polynomial 2°, linear, power (power term constrained to ≥ 1), and exponential models (degrees 3 and 5) were selected for each probe based on the lowest Akaike Information Criterion. A benchmark response of 1 standard deviation was selected. Probes with the following criteria were removed: (1) having a BMC greater than the highest concentration used in the analysis after excluding cytotoxic or non-soluble concentrations; (2) mapping to more than one gene; (3) having a model fit p-value < 0.1 determined by a likelihood ratio test; (4) having a BMC to BMC lower (BMCL) ratio > 20; (5) having a BMC upper (BMCU) to BMCL ratio > 40. Probes that met all the BMC filtering criteria were converted to their corresponding Entrez Identifiers. Data were further analyzed using R statistical software (version 4.3.2). Gene accumulation plots were generated by rank ordering genes fitting BMCs (lowest to highest) and plotting the BMC of each gene on the x-axis and rank along the y-axis.

Two types of tPODs were produced that we called: (1) general toxicity; and (2) ERα-specific response.

Three methods were used to derive general toxicity tPODs:

- 25th rank-ordered gene BMC: Genes fitting BMCs were rank ordered from lowest to highest BMC and the BMC for the 25th gene was selected as an early transcriptomic tPOD as previously described (Reardon et al. 2023; Matteo et al. 2023, 2025).
- Lowest pathway BMC: The lowest pathway tPOD was derived by aligning genes and their associated BMCL/BMC/BMCU values to the gene sets in Reactome Pathways database (https://reactome.org/) or the Kyoto Encyclopedia of Genes and Genomes (KEGG; https://www.genome.jp/kegg/). This approach is similar to the one recommended by the US National Toxicology Program (National Toxicology Program 2018). Gene sets that contained at least three genes fitting BMCs (genes that pass all criteria in the analysis), were at least 5% populated (based on total annotated gene number), and contained at least 40 genes in the whole gene set were selected. The lowest 5th percentile BMC of the selected gene sets were selected as tPODs.
- Lowest Consistent Response Dose (LCRD): We applied an approach previously described (Crizer et al. 2021) to identify the lowest concentration at which a consistent change in biological activity begins to occur.

We derived ERα-specific tPODs by calculating the 5th percentile BMC of 50 genes that comprise a published ERα biomarker (Corton et al. 2024b). We first confirmed that a chemical activated the ERα biomarker for at least one exposure concentration as described above (see Gene expression biomarker analysis). We filtered by (a) requiring a minimum of three genes with BMCs in the biomarker gene set to derive an ERα BMC and (b) that those genes have rank ordered BMCs below 200.

For all tPODs, confidence intervals for gene sets were estimated using a parametric bootstrap where the residuals were assumed to be normally distributed. For each gene, 100 bootstrap samples were generated followed by BMC analysis. Experiments were simulated using the bootstrapped results to obtain a BMC distribution. Experiment inclusion probabilities for each gene were estimated using the proportion of bootstrap samples that resulted in a BMC. For each simulated experiment, a uniform random number between 0 and 1 was generated for each gene. If the random number was less than the gene’s inclusion probability, the gene was retained and a BMC from the list of estimated BMCs for that gene was randomly selected. The median value from each of the 10,000 simulated experiments was used to estimate the tPOD median. The 95% confidence interval was estimated using the 2.5th and 97.5th percentiles from the simulated distribution.

### Ingenuity pathway analysis

Ingenuity Pathway Analysis (IPA; QIAGEN, Redwood City, CA, USA) was used to identify changes to upstream regulators and canonical pathways as previously described (Matteo et al. 2023, 2025). For each chemical tested, Excel files were imported into IPA containing gene IDs (Gene Symbol and Entrez ID) for the genes with a BMC (i.e., passed all filtering criteria), as well as their Williams trend test p-values from the BMC analysis, and the largest fold-changes of the gene relative to solvent controls (exported filtered data from BMDExpress3). This approach allowed us to analyze for the enrichment of genes showing robust concentration-responses to the exposures. IPA Core Analysis with a gene expression threshold of absolute fold change ≥ 1.5 and false discovery rate (FDR) adjusted *p* value ≤ 0.05 was used with the direct and indirect relationship settings based on experimental and highly predicted data. Statistical significance of the overlap (FDR-adjusted *p* value ≤ 0.05) between the data set and known targets of upstream regulators in IPA were calculated using Fisher’s Exact tests. The z-score was calculated using Fisher’s Exact Test based on the expected relationship for directions between upstream regulators and target genes and those observed in the data set. The following filters were used: p-value ≤ 0.05, z-score ≥ 1.5 or ≤ -1.5, Tissue & Cell Lines (breast cancer cells: MCF-7). A z-score of ≥ 2 (activated) or ≤ -2 (inhibited) was considered statistically significant.

## Results

### Cell viability

Cell viability was assessed after 48 h of chemical exposure using the CellTiter-Blue Cell Viability assay. The following conditions were cytotoxic and removed from the analysis: AO425 (50 µM), AO2246 (50 µM), BPK (50 µM), BPM (50 µM), DA-BPA (50 µM), PA08 (50 µM), THPE (10, 50 µM) (Supplementary Fig. 1).

### Transcriptomic data summary

The median read depth was approximately 2 million per sample with a median range of 91.8% mapped reads. Of 512 samples, 26 experimental samples were lost to QC, along with 24 reference RNA and lysis buffer control samples (see Supplementary File 1). The total number of samples per condition is listed in Supplementary Table 1.

The total number of genes fitting BMCs for each chemical tested is shown in Table 2. All chemicals tested were transcriptionally active and most had 200 – 600 genes fitting BMCs. DA-BPA (515), AO2246 (504), and PA08 (431) were the chemicals with the most genes fitting BMCs; BTH (235), BPM (235), AO425 (234), and PRed (87) had the least. The gene accumulation plot shows the concentrations at which the first 100 genes fitting BMCs were affected for each chemical tested (Fig. 3).

**Table 2:** Comparison of transcriptomic points of departure (tPODs)

Chemical	# Genes fitting BMCs	25th gene BMC	25th gene BMCL–BMCU	Lowest pathway 5th percentile BMC	LCRD	# Genes fitting ERα biomarker	ERα 5th percentile BMC	ERα 5th percentile BMCL–BMCU
BPK	383	0.036	0.0053–0.10	0.010	0.0075	11	0.0099	0.00091–0.034
PA08	431	0.057	0.041–0.11	0.048	0.016	7	0.0070	0.0011–0.024
BPA	388	0.14	0.048–0.35	0.11	0.085	21	0.10	0.029–0.26
BPE	345	0.15	0.052–0.33	0.18	0.0032	20	0.10	0.040–0.29
AO425	234	0.18	0.11–0.50	0.53	0.012	2		
BTH	235	0.23	0.13–1.06	0.23	0.010	2		
DA-BPA	515	0.29	0.14–0.61	0.17	0.099	16	0.44	0.31–0.78
BPM	235	0.36	0.26–0.56	0.084	0.063	7	0.42	0.29–0.72
THPE	357	0.53	0.35–1.03	0.58	0.12	9	1.69	1.29–2.46
AO2246	504	0.96	0.50–5.77	1.01	0.050	2		
TBBPS	426	9.71	5.45–12.84	9.71	1.99	2		
TBPB	273	20.54	10.13–40.77	5.87	5.81	2		
PRed	87	32.38	20.82–72.80		6.19	2		

tPODs were derived by prefiltering data using the Williams trend test (*p* < 0.05) and an absolute fold-change filter of ≥ 1.5 and postfiltered with the following settings in BMDExpress v3: Best benchmark concentration (BMC)/benchmark concentration lower (BMCL) < 20, Best BMC upper (BMCU)/BMCL < 40, and Best fitPvalue ≥ 0.1. tPODs (shown in µM) representing the 25th rank ordered gene benchmark concentration (BMC), the lowest pathway 5th percentile BMC, the lowest consistent response dose (LCRD), and the 5th percentile gene BMC for the estrogen receptor alpha (ERα) biomarker gene set are shown. BMCL and BMCU are used for the lower and upper bounds, respectively. Chemicals are shown in decreasing order of potency based on tPODs from the 25th gene BMC

### Transcriptomic biomarker activation

We used published transcriptomic biomarkers to explore general cellular stress responses following exposure to chemicals. The biomarkers were specific to: (a) NRF2 (Rooney et al. 2020); (b) HSF1 (Cervantes and Corton 2021); (c) MTF1 (Jackson et al. 2020); (d) TGx-DDI (Li et al. 2019); (e) TGx-HDACi (Cho et al. 2021); (f) NF-kB (Korunes et al. 2022); (g) HIF1α (Corton et al. 2025); (h) cellular proliferation (Corton et al. 2024a); (i) x-box binding protein 1 (XBP1) (manuscript in preparation). As in our previous work (Matteo et al. 2023, 2025), we considered activation of at least two of these biomarkers within a condition as an indication of overt cellular stress (Escher et al. 2020) and removed these concentrations from subsequent BMC analyses. Supplementary File 2 provides a full list of all stress response biomarkers activated and inhibited.

**Fig. 2:**
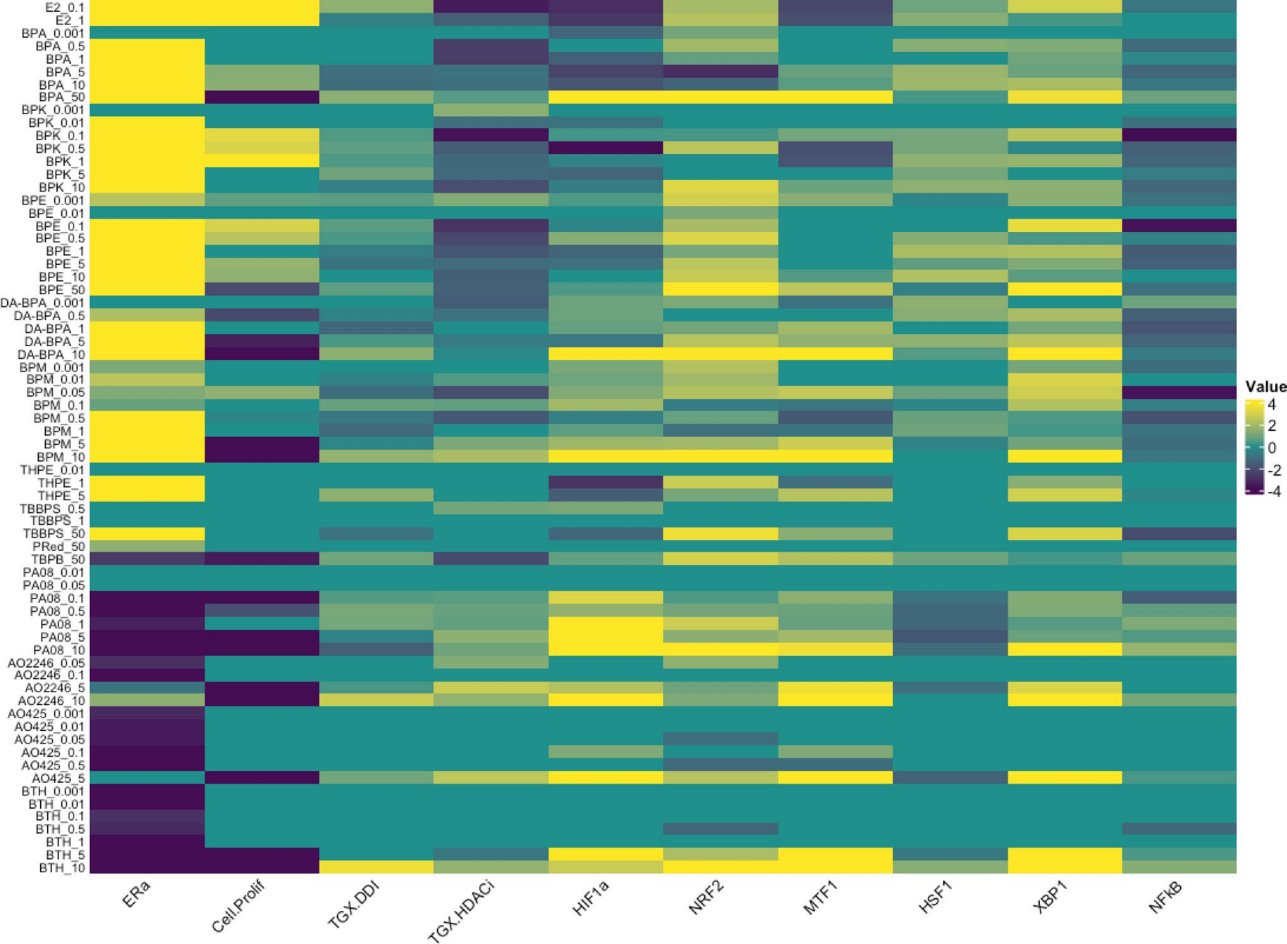
Summary of transcriptomic biomarker activity. The biomarkers are used to detect chemicals that perturb the estrogen receptor alpha (ERa), affect cellular proliferation (Cell.Prolif), cause DNA damage (TGX.DDI), affect histone deacetylases (TGX.HDACi), affect cellular stress response pathways (HIFa, NRF2, MTF1, HSF1, XBP1, NFkB). The biomarker was activated (yellow) or inhibited (blue) based on thresholds for significance − log(*p* value) ≥ 4 or ≤  − 4 (color figure online)

Most plastics-related chemicals activated stress response biomarkers at the highest exposure concentrations tested (Fig. 2, Supplemental File 2). Most chemicals inhibited the cellular proliferation biomarker: AO425 (5 µM), AO2246 (5, 10 µM), BPA (50 µM), BPM (5, 10 µM), BTH (5, 10 µM), DA-BPA (10 µM), PA08 (0.1, 5, 10 µM). Only BPK (1 µM) and the positive control (E2; 0.1, 1 nM), activated the cellular proliferation biomarker in this model. Most chemicals activated the HIFα biomarker: AO425 (5 µM), AO2246 (10 µM), BPA (50 µM), BPM (10 µM), BTH (5 µM), DA-BPA (10 µM), PA08 (1, 5, 10 µM). Only BPK (0.5 µM) inhibited this biomarker. Half of chemicals tested activated the NRF2 biomarker: BPA (50 µM), BPE (50 µM), BPM (10 µM), BTH (10 µM), DA-BPA (10 µM), PA08 (10 µM). Many of the chemicals activated the MTF1 biomarker: AO425 (5 µM), AO2246 (10 µM), BPA (50 µM), BPM (10 µM), BTH (5, 10 µM), DA-BPA (10 µM). Most of the same chemicals also activated the XBP1 biomarker: AO425 (5 µM), AO2246 (10 µM), BPE (50 µM), BPM (10 µM), BTH (5, 10 µM), DA-BPA (10 µM), PA08 (10 µM).

Based on these data, the following conditions were considered overtly cytotoxic and removed from further analyses: AO425 (5 µM), AO2246 (10 µM), BPA (50 µM), BPE (50 µM), BPM (10 µM), BTH (5, 10 µM), DA-BPA (10 µM), PA08 (10 µM).

### ERα biomarker activation

We used a 50 gene transcriptomic ERα biomarker (Corton et al. 2024b) to test the ability of chemicals to activate/inhibit the ERα.

Most BPA-like plastic-related chemicals activated the ERα biomarker: BPA (0.5, 1, 5, 10, 50 µM), BPE (0.1, 0.5, 1, 5, 10, 50 µM), BPK (0.01, 0.1, 0.5, 1, 5, 10), BPM (0.5, 1, 5, 10 µM), DA-BPA (1, 5, 10 µM), THPE (1, 5 µM), TBBPS (50 µM). Importantly, E2 (0.1, 1 nM), the positive control, activated the ERα biomarker. Several chemicals tested inhibited the ERα biomarker: AO425 (0.1, 0.5 µM), AO2246 (0.1 µM), PA08 (0.1, 0.5, 5, 10 µM), BTH (0.001, 0.01, 1, 5, 10 µM).

### Transcriptomic point of departure (tPOD) derivation

We used two approaches to generate tPODs based on either (a) general toxicological effects or (b) ERα activation (mode of action-specific).

#### Potency ranking for general toxicity

**Fig. 3:**
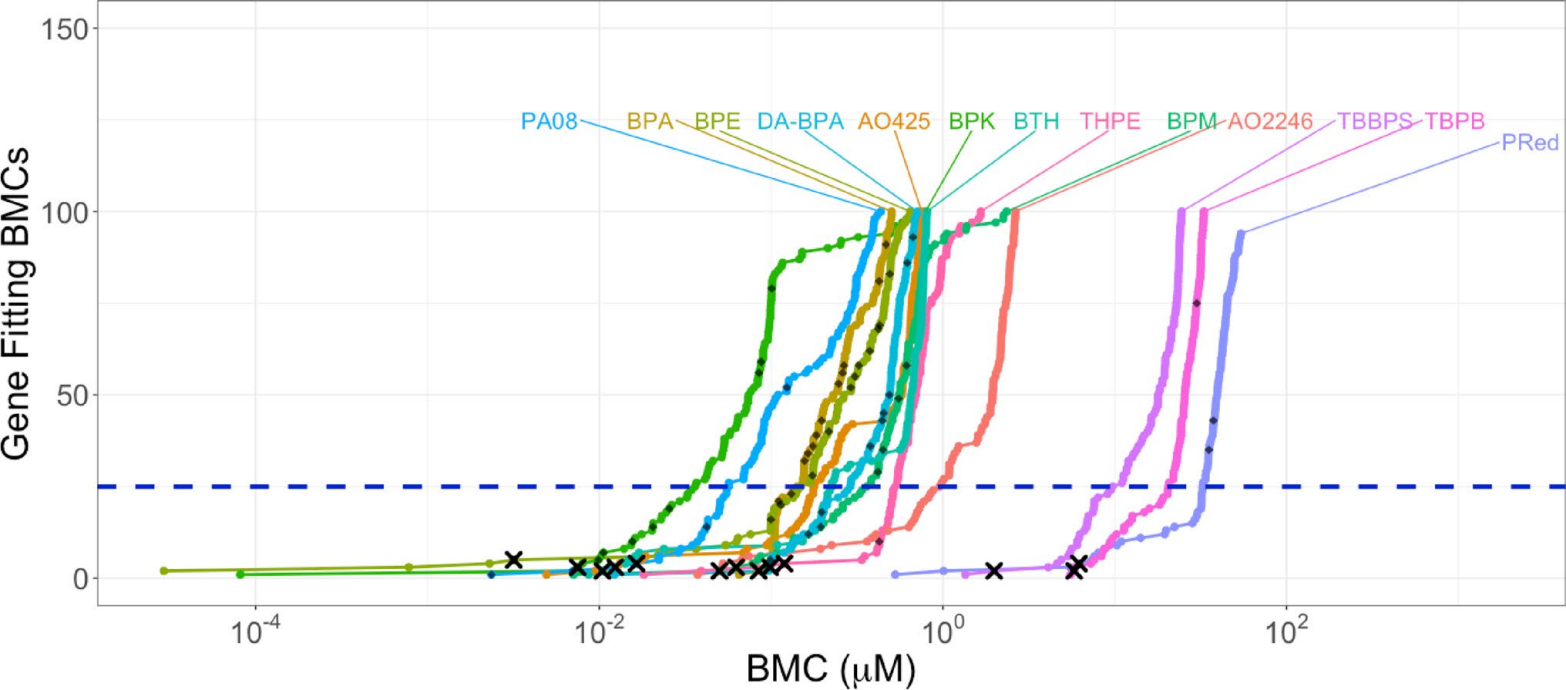
Gene accumulation plot of first 100 genes fitting benchmark concentrations (BMCs). Data were prefiltered using the Williams trend test (*p* < 0.05) and an absolute fold-change filter of ≥ 1.5, and post filtered with the following settings in BMDExpress v3: Best benchmark concentration (BMC)/benchmark concentration lower (BMCL) < 20, Best BMC upper (BMCU)/BMCL < 40, and Best fitPvalue ≥ 0.1 The lowest consistent response dose (LCRD; black X’s) and genes fitting the estrogen receptor alpha (ERα; black diamonds) are highlighted. Blue horizontal dashed line denotes the 25th rank order gene BMC (color figure online)

We derived general toxicity tPODs to identify concentrations producing a ‘concerted molecular change’ (Johnson et al. 2022). The gene accumulation plot shows the rank order of the potency of the chemicals tested based on the 25th gene BMC (Fig. 3), from most to least potent (left to right side of graph). BPK was the most potent chemical tested, followed by PA08, and BPA (Fig. 3).

We used three approaches to identify exposure concentrations that cause a robust change in transcriptomic activity: (1) the 25th gene BMC, (2) the lowest pathway gene set 5th percentile BMC, and (3) the LCRD.

**Fig. 4:**
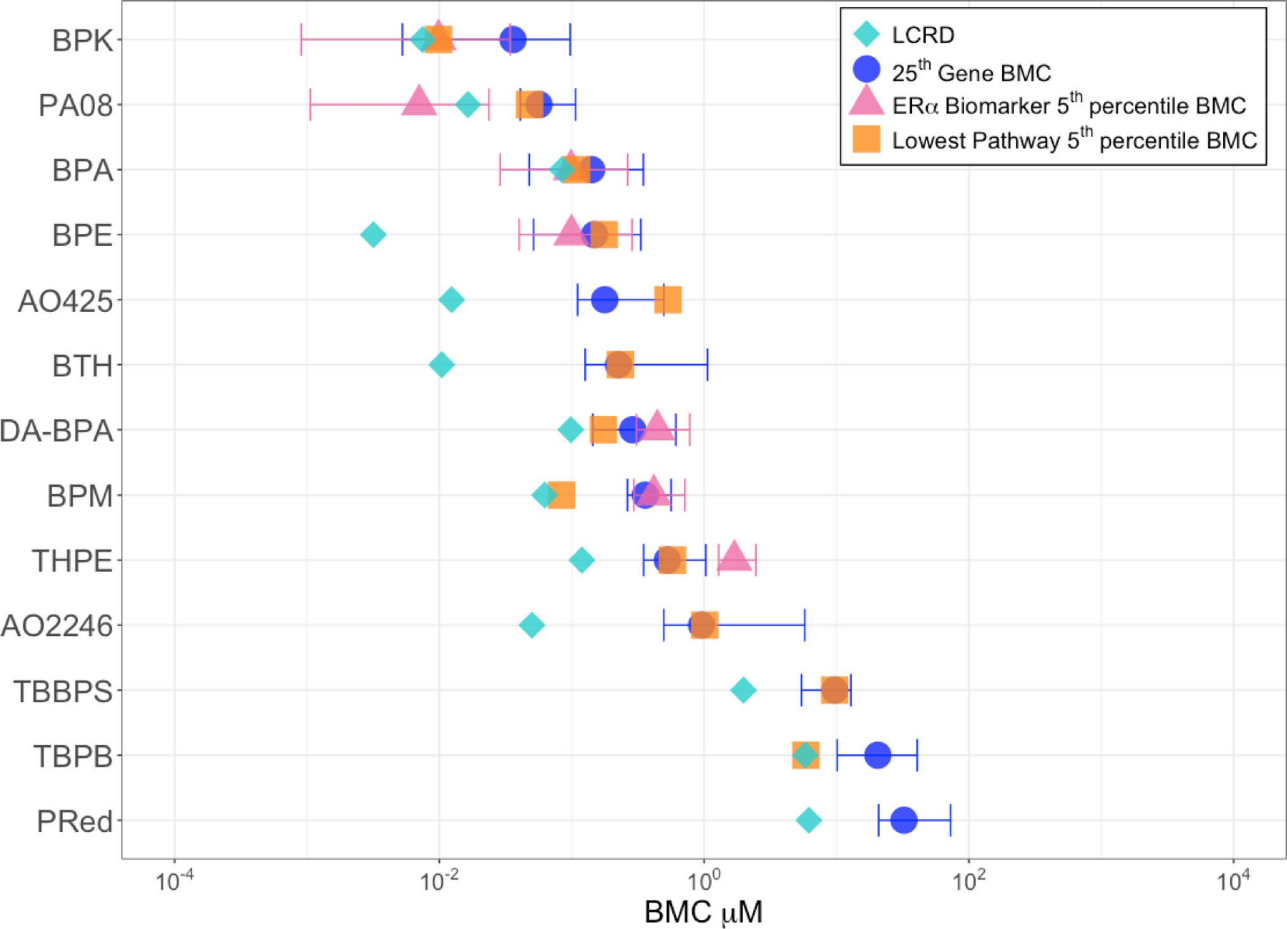
Comparison of transcriptomic points of departure (tPODs) between chemicals tested. tPODs were derived by prefiltering data using the Williams trend test (*p* < 0.05) and an absolute fold-change filter of ≥ 1.5 and postfiltered with the following settings in BMDExpress v3: Best benchmark concentration (BMC)/benchmark concentration lower (BMCL) < 20, Best BMC upper (BMCU)/BMCL < 40, and Best fitPvalue ≥ 0.1. tPODs (shown in µM) representing the LCRD, 25th rank ordered gene benchmark concentration (BMC), the lowest pathway 5th percentile BMC, and the 5th percentile gene BMC for the estrogen receptor alpha (ERα) biomarker gene set are shown. BMC lower (BMCL) and BMC upper (BMCU) are used for the lower and upper bounds, respectively. Chemicals are shown in decreasing order of potency based on tPODs from the 25th gene BMC

The 25th gene BMC identifies the point at which transcriptional changes begin, filtering out genes within background variability and biologically inactive chemicals (see Fig. 3). Most chemicals tested had 25th gene BMCs between 0.1 and 10 µM (Fig. 4, Table 2). BPK had the lowest 25th gene BMC (0.036 µM), followed by PA08 (0.057 µM), and BPA (0.14 µM); TBBPS (9.71 µM), TBPB (20.54 µM), and PRed (32.38 µM) had the highest 25th gene BMCs.

The 5th percentile BMC of the lowest pathway provides a conservative estimate of the most potent gene set-level biological response. The tPODs derived using this approach were highly similar to the 25th gene (Fig. 4, Table 2). BPK had the lowest 5th percentile lowest pathway BMC (0.010 µM), followed by PA08 (0.048 µM), and BPM (0.084 µM). AO2246 (1.01 µM), TBPB (5.87 µM), and TBBPS (9.71 µM) had the highest 5th percentile lowest pathway BMC.

The LCRD represents the most sensitive non-outlier concentration where a consistent biological response begins. For most chemicals tested, the LCRD was one order of magnitude lower than the 25th gene and the pathway BMC (Fig. 4, Table 2). BPE (0.0032 µM), BPK (0.0075 µM), and BTH (0.010 µM) had the lowest LCRD. TBBPS (1.99 µM), TBPB (5.81 µM), and PRed (6.19 µM) had the highest LCRD.

#### Potency ranking based on the ERα biomarker

To determine the potencies of the chemicals in activating the ERα, an ERα-specific tPOD was calculated as the 5th percentile of BMCs from the ERα biomarker gene set (Corton et al. 2024b), after confirming biomarker activation and applying minimum gene and BMC filters (Table 2, Fig. 4). Seven chemicals including BPA, BPK, PA08, BPE, DA-BPA, BPM, and THPE passed these criteria. Most ERα biomarker genes were activated at higher concentrations than the 25th gene, but within the first 200 genes fitting BMCs (Supplementary Fig. 2). Most chemicals tested had an ERα 5th percentile BMC between 0.10 and 1.7 µM. BPK had the lowest ERα 5th percentile BMC (0.010 µM), followed by BPA (0.10 µM), and BPE (0.10 µM); BPM (0.42 µM), DA-BPA (0.44 µM), and THPE (1.69 µM) had the highest.

### Ingenuity pathway analysis

To determine the primary mechanisms altered by the chemicals, we used the genes fitting BMCs, their maximum fold changes, and their p-values, in an IPA canonical pathway and upstream regulator analysis as described previously (Matteo et al. 2023, 2025). A full list of canonical pathways and upstream regulators affected by chemical exposure is available in Supplementary File 3.

**Fig. 5:**
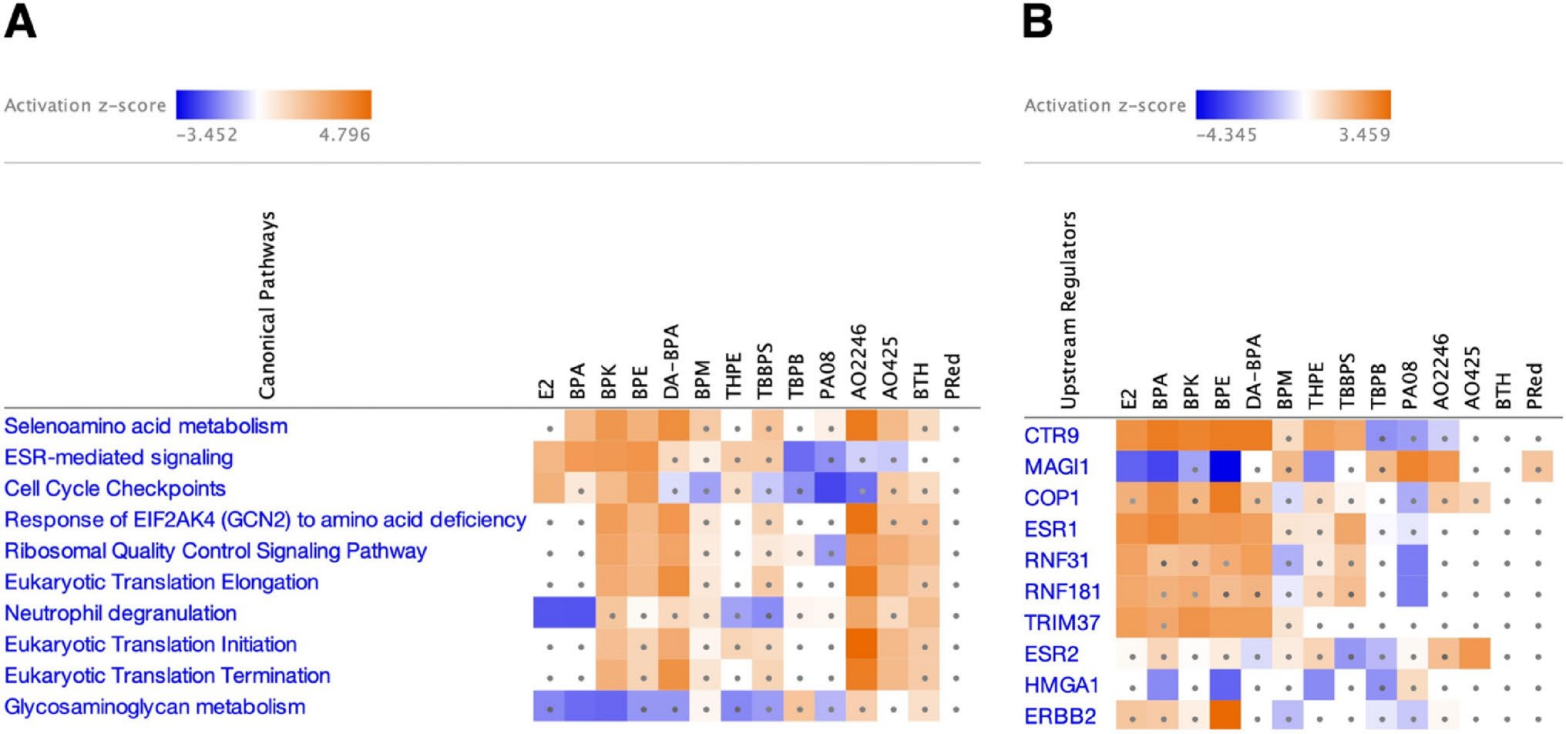
Ingenuity Pathway Analysis. A list of genes fitting benchmark concentration models, the Williams trend test *p* value, and the maximum fold-change were imported into IPA. Filters were set to |z-score ≥ 2.0| and *p* value < 0.05. Orange denotes predicted activation and blue predicted inactivation (dots signify |z-score < 2|). **A** top 10 canonical pathways; **B** top 10 upstream regulators (color figure online)

Most chemicals tested activated canonical pathways (Fig. 5A) associated with amino acid metabolism (selenoamino acid metabolism, response of EIF2AK4 (GCN2) to amino acid deficiency), cellular stress (ribosomal quality control signaling pathway), or translation (translation elongation, translation initiation, translation termination). Further, BPA and most BPA-like plastic-related chemicals tested activated pathways associated with the estrogen receptor (ESR-mediated signaling) and cell cycle control (cell cycle checkpoints). By contrast, a dye (TBPB) and several other chemicals in plastics were predicted to inhibit the ER and cell cycle pathways. These data align with results from ERα and cellular proliferation biomarkers, respectively (see Fig. 2). Finally, most chemicals tested were predicted to inhibit a pathway related to carbohydrate metabolism (glycosaminoglycan metabolism).

BPA-like plastic-related chemicals, including BPK, BPE, and DA-BPA, activated similar upstream regulators as the positive control E2 and BPA (Fig. 5B). These include upstream regulators associated with the estrogen receptor (ESR1), transcriptional regulation (CTR9, HMGA1), cell–cell adhesion (MAGI1), and ubiquitination (COP1, RNF31, RNF181, TRIM37). Other chemicals like BPM and THPE also perturbed similar upstream regulators as BPA and E2, although enrichment was generally weak. By contrast, TBPB, and other chemicals with different features relative to BPA had opposite enrichment to BPA-like chemicals, but most predictions did not reach significance. The dye PRed was not predicted to significantly enrich any gene sets.

## Discussion

Plastics remain an important material due to their functionality, durability and cost-effectiveness, and are prominently used in almost all aspects of modern living. As a result, humans may be exposed to chemicals in plastics throughout daily life, yet many remain data-poor and lack toxicological assessments. To address these knowledge gaps, we applied HTTr to screen 9 chemicals in plastics, an antimicrobial, as well as two dyes used in laboratory settings and some consumer products in MCF-7 breast cancer cells, building on our previous work (Matteo et al. 2023, 2025). We derived general, pathway-, and ERα-specific measures of potency to compare chemicals to an established endocrine disruptor, BPA. Most plastic-related chemicals, along with the antimicrobial tested, had similar general toxicological potencies in vitro. In contrast, transcriptomic biomarker analysis identified that most BPA-like chemicals tested activated the ERα biomarker, whereas plastic chemicals with distinct chemical features compared to BPA (i.e., non-BPA-like) inhibited the ERα biomarker. Pathway and upstream regulator analyses highlighted that BPA-like chemicals in plastics had similar gene expression profiles, while non-BPA-like chemicals had broadly opposing directionality in gene enrichment. Together, these data support that the plastics-related chemicals tested have similar toxicological potencies, and that they possibly have opposite effects on the ERα activity in vitro.

Most BPA-like chemicals tested activated the ERα transcriptomic biomarker in MCF-7 cells, consistent with prior HTTr studies from our group (Matteo et al. 2023, 2025) and others (Beal et al. 2024) applying the same biomarker (Corton et al. 2024b). In contrast, most non-BPA-like chemicals inhibited the biomarker indicative of ER antagonism. Many of these chemicals that exhibited ER antagonism (BTH, AO425, AO2246) feature hydroxyl groups in the orthogonal position of the aromatic rings, whereas BPA and many of its analogues are bisphenols with para-hydroxyl substitutions, suggesting that structural orientation influences chemical activity on ERα in vitro. To our knowledge, data on ERα activation for many of the chemicals in plastics tested are not publicly available, although some structurally related compounds have been studied. For example, BTH resembles triclosan, an antibacterial reported as ERα active (Gee et al. 2008), yet in this study BTH inhibited the biomarker. The brominated flame retardant TBBPS was only ERα active at the highest concentration tested and failed to meet the minimum gene-response criteria for classification as ERα active. This result is consistent with its analogue TBBPA, which is also ERα inactive in MCF-7 cells (Pelch et al. 2019). PRed was also ERα inactive, despite early MCF-7 studies reporting ERα binding (Berthois et al. 1986; Welshons et al. 1988), warranting follow-up to determine whether binding translates to functional activity. This may be due to the sulfone bridge on PRed makes it bulkier and rigid unlike the isopropylidene group of BPA that makes it more flexible. Finally, TBPB was the most structurally distinct compound relative to BPA and did not activate ERα, consistent with reports that polybrominated phenols generally exhibit weak or negligible ERα binding (Olsen et al. 2002). Collectively, these results support that most BPA-like structures act as ERα agonists in vitro, while the non-BPA-like chemicals in plastics tested are mostly predicted to behave as antagonists.

We applied tPODs based on analysis of general, pathway-, and ERα-specific measures of gene expression to compare the toxicological potency of chemicals in MCF-7 cells. Most chemicals tested had 25th gene BMCs within one order of magnitude of each other, and LCRD, pathway- and ERα-specific tPODs produced similar potency rankings. LCRDs were generally lower than other tPODs by an order of magnitude, in line with a previous HTTr experiment in MCF-7 cells by our lab (Matteo et al. 2025), likely because the LCRD captures the genes at the low end of an ordered gene BMC distribution. This makes it inherently more sensitive than tPODs derived from enriched gene sets or summary statistics of broader dose‑responsive gene pools. BPA-like chemicals tended to be more potent than the structurally distinct chemicals in plastics, likely reflecting differences in ERα activity. BPK was the most potent chemical tested across general and ERα-specific tPODs. Interestingly, PA08 was the second most potent overall, despite its high molecular weight and putative ERα antagonistic properties. This finding challenges the conventional assumption that high molecular weight chemicals are biologically inert (Groh et al. 2022) and suggests that molecular size alone does not preclude transcriptional activity. To our knowledge there is little publicly available information on PA08 in vitro. BPA, the most well studied chemical of its class, was the third most potent and had similar tPODs as previous HTTr experiments in MCF-7 cells (Beal et al. 2024; Matteo et al. 2025). The least potent chemicals (TBBPS, PRed, and TBPB) were those that failed to activate ERα. It is possible that these chemicals may exhibit different toxicological potencies in cell models that do not overexpress ERα. Overall, these findings show that the plastic-related chemicals tested are bioactive in MCF-7 cells with similar potencies and underscore the value of using multiple tPODs to rank chemical potencies in HTTr studies as previously demonstrated across chemical classes (Reardon et al. 2023).

Transcriptomic biomarkers were used to identify chemical exposure concentrations that caused cellular stress responses in vitro. Most chemicals tested perturbed multiple biomarkers in MCF-7 cells. Specifically, many of these chemicals (e.g., AO2246, AO425, BPA, BPM, BTH, DA-BPA, PA08) activated the HIF1α, NRF2, MTF1, and/or XBP1 biomarkers, as well as inhibiting the cellular proliferation biomarker at the highest exposure concentrations tested, consistent with cellular stress responses and previous HTTr screening studies of plastics-related chemicals in MCF-7 cells (Matteo et al. 2023, 2025). Notably, PA08 activated the HIF1α biomarker at the top three exposure concentrations, suggesting that this chemical may increase survival potential of cancer cells in hypoxic conditions (Thomas and Kim 2008). MTF1 is a transcription factor that has been associated with cancer progression in certain malignancies (Zhang et al. 2024a), as well as an indicator of stress response, which seems to be the case for these chemicals which activated the biomarker only at high concentrations. However, an important limitation is that these transcriptomic biomarkers were developed using multiple cell lines and thus, these data would benefit from validation in other in vitro systems. Overall, these data support that high exposure concentrations of plastics-related chemicals activate cellular stress response pathways in MCF-7 cells.

Genes with modeled BMCs were analyzed using Ingenuity Pathway Analysis to identify potential mechanisms of action in MCF-7 cells. Most BPA-like plastic-related chemicals perturbed similar pathways associated with ERα signaling, amino acid metabolism, cellular stress responses, and translation. Upstream regulator analysis showed strong overlap between BPA-like chemicals and the positive control E2, reinforcing the similarity in their mechanisms of action in breast cancer cells that overexpress the ERα. These findings are also consistent with our previous HTTr studies in MCF-7 cells (Matteo et al. 2023, 2025), highlighting the reproducibility of in vitro transcriptomic signatures across HTTr studies. In contrast, many of the non-BPA-like chemicals enriched relatively few gene sets, likely reflecting their lower bioactivity in MCF-7 cells. However, MCF-7 cells are highly sensitive to estrogenic chemicals and thus it is possible that non-ERα mechanisms are underrepresented in the cell system. Overall, very little is currently known about the mechanisms of action of these plastic chemicals. For example, one study identified more than 150 potentially carcinogenic plastic-related chemicals among ~ 2,000 screened (Vincoff et al. 2024). Similarly, the high molecular weight chemicals evaluated perturbed few pathways or upstream regulators, in line with the transcriptomic biomarker results, suggesting that these compounds drive largely non-specific transcriptomic responses in MCF-7 cells. Collectively, these results support grouping structurally similar chemicals to BPA for read-across applications and emphasize the need for further investigation into the mechanisms of action of chemicals in plastics in vitro.

In summary, these HTTr data support that many of the chemicals in plastics tested act through the ERα in vitro. BPA-like plastic-related chemicals consistently activated the ERα biomarker, whereas many of the non-BPA-like chemicals appeared to antagonize the receptor. Potency rankings were similar across general, pathway-, and ERα-specific measures, with BPK emerging as the most potent chemical tested, activating ERα at the lowest concentrations, followed by PA08 and BPA. In addition to ERα signaling, BPA-like chemicals in this study perturbed pathways related to translation and cellular stress responses, while the other chemicals tested produced weaker or opposite gene expression profiles. Together, these results highlight the reproducibility of HTTr across studies and underscores its utility for grouping BPA-like chemicals, investigating poorly characterized chemicals in plastics, and providing new information to support human health risk assessment activities.

## Supplementary Information

Below is the link to the electronic supplementary material.

Supplementary Material 1: Samples removed due to R-ODAF filtering criteria

Supplementary Material 2: Summary of perturbed stress response biomarkers

Supplementary Material 3: List of perturbed IPA canonical pathways and upstream regulators

Supplementary Material

## Data Availability

Data are available through the NCBI Gene Expression Omnibus with GEO accession GSE312456.
