# Supplementary material for: Chemicals in plastics differentially affect the transcriptome of MCF-7 breast cancer cells
Source: Arch Toxicol. 2026 Apr 20;100(7):3011–24. doi: 10.1007/s00204-026-04376-1 (PMC13309450; doi:10.1007/s00204-026-04376-1)
Supplement: Supplementary file 4 — Supplementary Material [file 204_2026_4376_MOESM4_ESM.docx]

**Title:** Chemicals in plastics differentially affect the transcriptome of MCF-7 breast cancer cells

**Authors:**

Geronimo Matteo^1,2^, ORCID: 0000-0003-0819-4471

Dave C. Eickmeyer^2^, ORCID: 0000-0001-5434-2565

Lauren M. Bradford^1^, ORCID: 0000-0003-0686-1648

Matthew J. Meier^1^, ORCID: 0000-0001-8199-8754

Andrew Williams^1^, ORCID: 0000-0002-7637-7686

Tara Barton-Maclaren^3^, ORCID: 0000-0002-2929-7747

J. Christopher Corton^4^, ORCID: 0000-0002-6197-2036

Carole L. Yauk^2^, ORCID: 0000-0002-6725-3454

Ella Atlas^1,5^, ORCID: 0000-0003-4410-4402

**Corresponding Author**: Dr. Ella Atlas, 251 Sir Frederick Banting Driveway, Ottawa, ON, K1A 0K9, ella.atlas@hc-sc.gc.ca

**Affiliations**:

^1^ Environmental Health Science and Research Bureau, Health Canada, Ottawa, Canada
^2^ Department of Biology, University of Ottawa, Ottawa, Canada
^3^ Existing Substances Risk Assessment Bureau, Ottawa, Canada
^4^ Center for Computational Toxicology and Exposure, US Environmental Protection Agency, Research Triangle Park, USA
^5^ Department of Biochemistry, University of Ottawa, Ottawa, Canada

**Supplementary Table 1**: Total number of replicates per condition included in the analysis

| Chemical | Concentration (µM) | | | | | | | | | |
| --- | --- | --- | --- | --- | --- | --- | --- | --- | --- | --- |
|  | 0.0001 | 0.001 | 0.01 | 0.05 | 0.1 | 0.5 | 1 | 5 | 10 | 50 |
| E2 | 8 | 8 |  |  |  |  |  |  |  |  |
| BPK |  | 4 | 4 | 4 | 4 | 4 | 4 | 4 | 4 |  |
| PA08 |  | 4 | 4 | 4 | 4 | 4 | 4 | 4 |  |  |
| BPA |  | 4 | 3 | 4 | 4 | 4 | 4 | 4 | 4 |  |
| BPE |  | 3 | 4 | 4 | 4 | 4 | 4 | 4 | 4 |  |
| AO425 |  | 4 | 4 | 4 | 4 | 4 | 4 |  |  |  |
| BTH |  | 4 | 4 | 4 | 4 | 4 | 4 |  |  |  |
| DA-BPA |  | 4 | 4 | 4 | 4 | 4 | 4 | 4 |  |  |
| BPM |  | 3 | 4 | 4 | 4 | 4 | 4 | 4 |  |  |
| THPE |  | 4 | 4 | 4 | 4 | 4 | 4 | 4 |  |  |
| AO2246 |  | 4 | 4 | 4 | 4 | 4 | 4 | 4 |  |  |
| TBBPS |  | 4 | 4 | 4 | 3 | 4 | 4 | 4 | 4 | 3 |
| TBPB |  | 4 | 4 | 4 | 4 | 4 | 4 | 4 | 4 | 4 |
| PRed |  | 4 | 4 | 4 | 4 | 4 | 4 | 4 | 4 | 4 |


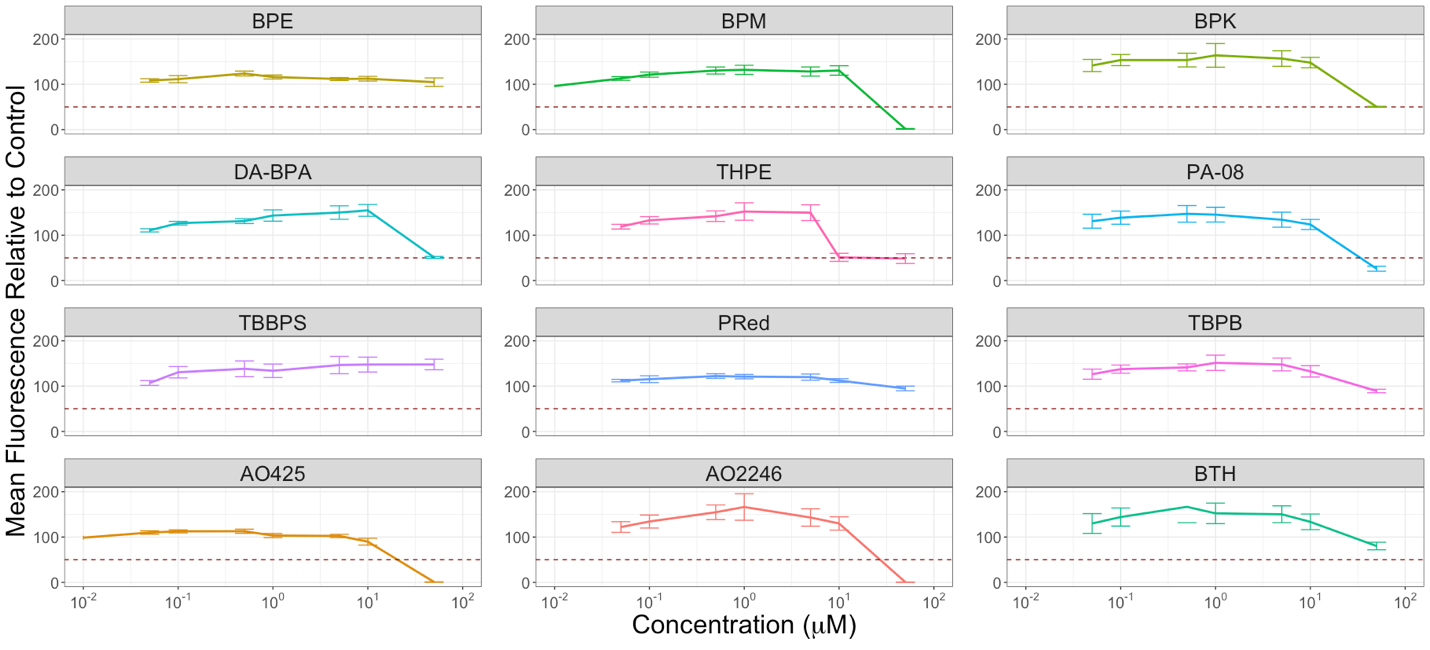


**Supplementary Figure 1**: Cell viability using a CellTiter-Blue Cell Viability Assay. MCF-7 cells (n = 3 – 4) were exposed to 10 plastic additives and two dyes at a range of concentrations (0.001 – 50 µM) for 48 h and are compared to their respective control (dimethyl sulfoxide; 0.1%) samples. First row of chemicals from left to right: BPE, BPM, BPK; second row: DA-BPA, THPE, PA-08; third row: TBBPS, PRed, TBPB; fourth row: AO425, AO2246, BTH. Blue horizontal dotted line denotes 50% cell viability relative to controls. The following conditions were considered cytotoxic: BPM (50 µM), BPK (50 µM), DA-BPA (50 µM), THPE (10, 50 µM), PA08 (50 µM), AO425 (50 µM), AO2246 (50 µM).


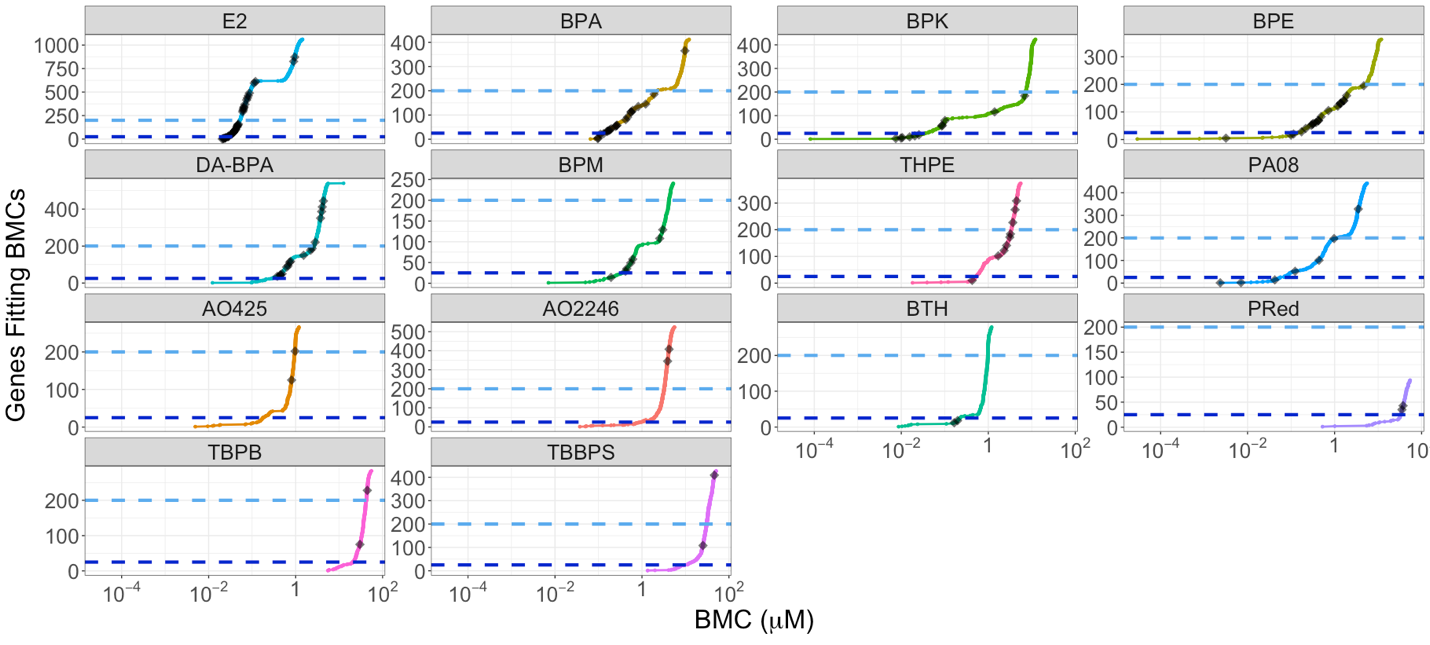


**Supplementary Figure 2**: Gene accumulation plot of MCF-7 cells (n = 3 – 4 per concentration) exposed 10 plastic additives, two dyes, and bisphenol A (BPA) at a range of concentrations (0.001 – 50 µM), as well as 17β-estradiol (E2; 0.1, 1 nM) for 48 h. Genes fitting the 50 gene estrogen receptor alpha biomarker are overlaid (black diamonds). The light blue dashed line indicates the 25^th^ ranked gene and the dark blue dashed line indicates the 200^th^ ranked gene. Data were prefiltered using the Williams trend test (p < 0.05) and an absolute fold-change filter of ≥ 1.5, and post filtered with the following settings in BMDExpress v3: Best benchmark concentration (BMC)/benchmark concentration lower (BMCL) < 20, Best BMC upper (BMCU)/BMCL < 40, and Best fitPvalue ≥ 0.1. First row of chemicals from left to right: E2, BPA, BPK, BPE; second row: DA-BPA, BPM, THPE, PA08; third row: AO425, AO2246, BTH, PRed; fourth row: TBPB, TBBPS.
